# How Does Self-Control Promote Health Behaviors? A Multi-Behavior Test of Five Potential Pathways

**DOI:** 10.1093/abm/kaac053

**Published:** 2022-10-07

**Authors:** Mark Conner, Sarah Wilding, Charles E Wright, Paschal Sheeran

**Affiliations:** School of Psychology, University of Leeds, Leeds, UK; School of Psychology, University of Leeds, Leeds, UK; Department of Psychology and Neuroscience, University of North Carolina at Chapel Hill, Chapel Hill, USA; Department of Psychology and Neuroscience, University of North Carolina at Chapel Hill, Chapel Hill, USA

**Keywords:** Reasoned action approach, Habits, Self-regulation, Willpower

## Abstract

**Background:**

Self-control is generally defined as the capacity to override impulses and is a robust predictor of health behaviors. This paper integrates trait, reasoned action, and habit approaches to develop and test a mechanistic account of *how* self-control influences health actions.

**Purpose:**

We tested five potential pathways from self-control to behavior, termed the *valuation, prioritization, habituation, translation*, and *inhibition* routes.

**Methods:**

At baseline, participants (*N* = 663 adults) completed survey measures of reasoned action approach variables and habits in relation to eight health behaviors and the Brief Self-Control Scale. Three months later, participants reported their behavior. Multi-level modeling was used to test pathways across behaviors.

**Results:**

Supporting the valuation route, affective attitude, cognitive attitude, descriptive norms, and perceived behavioral control mediated the self-control-intention relation, and intentions and perceived behavioral control mediated the relationship between self-control and health behaviors. Self-control also predicted the priority accorded to different considerations during intention formation. Higher self-control was associated with stronger prediction by cognitive attitudes and perceived behavioral control and weaker prediction by habits and injunctive norms. Self-control predicted habit formation, and habits mediated the self-control-behavior relation. Finally, self-control was associated with the improved translation of intentions into health behaviors and with greater inhibition of affective and habitual influences. Findings for the different pathways were not moderated by whether approach (health-protective behaviors) or avoidance responses (health-risk behaviors) were at issue.

**Conclusions:**

The present research offers new insights into why self-control promotes health behavior performance, and how deficits in self-control might be offset in future behavior-change interventions.

## Teaser

Individuals high in self-control do more health behaviors partly because they are better at following through on their intentions.

## Introduction

Trait self-control is the capacity to override impulses, resist temptations, and overturn dominant responses, to advance long-term over short-term goals, and to develop efficient, automatic processes that promote goal achievement [1–3]. Evidence indicates that self-control is stable over time [4], highly heritable [5], and not readily changed through training [6]. A meta-analysis by de Ridder et al. [1] found a small to medium-sized effect of self-control on goal attainment across 50 studies involving 15,455 participants (*r*_*+*_ = .26, 95%CI 0.23 to 0.28). Self-control was associated with both increases in desired or approach behaviors and with decreases in undesired or avoidance behaviors (*r*_*+*_ = .21 and −.23, respectively). The average correlation between self-control and dietary and weight outcomes was *r*_*+*_ = .17 across 14 studies [1], and other studies have reported relationships between self-control and exercise, sleep hygiene, fruit and vegetable intake, alcohol consumption, sexual risk-behavior, weight loss, and smoking (e.g., [7–11]; see [12] for review). Self-control, it appears, is a robust predictor of health behaviors.

How does self-control promote health behavior performance? Relatively little research has examined the mechanisms by which self-control influences behavior change (e.g., [13, 14]). Accordingly, we aimed to integrate and extend previous explanations of the impact of self-control on action (e.g., [9, 11, 14–16]). In particular, we specify five potential pathways from self-control to health behaviors and propose that higher self-control leads to: (i) setting more healthful goal intentions that increase behavioral performance (the *valuation* route); (ii) attaching greater weight to the utility, feasibility, and prescriptiveness of the action and less weight to affect and descriptive norms during intention formation (the *prioritization* route); (iii) forging habits that lead to efficient execution of health behaviors (the *habituation* route); (iv) improved capacity to turn healthful intentions into action (the *translation* route); and (v) greater success in overcoming affective or habitual influence (the *inhibition* route). We tested these routes in a longitudinal study of eight health behaviors.

Testing these pathways involves integrating trait self-control with two other theories of health behavior change, namely, the Reasoned Action Approach (RAA; [17]) and habit theory (e.g., [18]). The RAA is an extension of the Theory of Planned Behavior [19] that specifies intentions and perceived behavioral control as the proximal determinants of behavior, and attitudes, social norms, and perceived behavioral control as the determinants of intentions. Intentions index motivation to engage in a behavior [19]. Attitude is the person’s overall evaluation of the behavior and comprises beliefs about both instrumental consequences of acting (e.g., health benefits) and feelings that accrue from behavioral performance (termed *cognitive* and *affective attitudes*, respectively; [20, 21]). Social norms comprise both injunctive norms (perceived social pressure to perform a behavior) and descriptive norms (beliefs about rates of behavioral performance among one’s reference group) (e.g., [22]). Perceived behavioral control is the person’s appraisal of the ease or difficulty of acting or confidence in one’s ability to perform the behavior. Although perceived behavioral control may be subdivided into capacity and autonomy, only the capacity component predicts intention and behavior [23]. Meta-analysis indicates that affective attitudes, cognitive attitudes, injunctive norms, descriptive norms, and perceived behavioral control each predict health-related intentions and that intentions and perceived behavioral control predict health behaviors [23]. The RAA holds that variables such as self-control influences behavior by changing components of the RAA or moderating the relationship between these components and intention and/or behavior [17] and similar arguments may apply in relation to habit [18].

Habit theory (e.g., [18, 24]) was developed to explain the fact that much of human behavior involves actions that are repeatedly performed in the same contexts (e.g., at similar times and in similar places). According to the theory, behaviors that are frequently and consistently performed in the same circumstances come under the control of relevant situational cues that automatically elicit responses (e.g., [25]). Evidence indicates that measures of habit that tap performance frequency × context stability better predict health behaviors compared to intentions, and that habits moderate the influence of intentions on behavior (e.g., [26–27]).

**Fig. 1.**
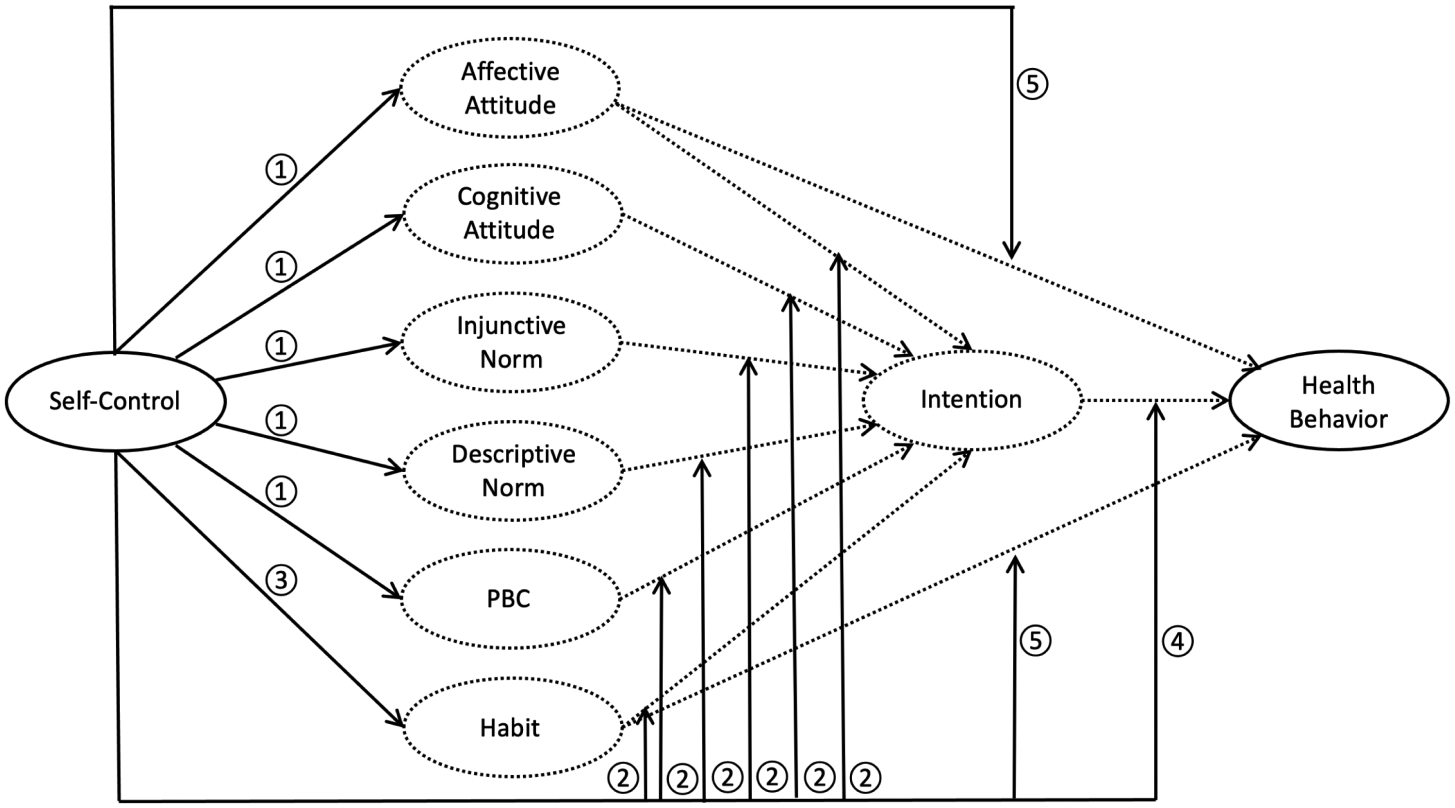
Potential pathways of influence from self-control to health behaviors. *Notes.* Dashed lines indicate paths specified by the reasoned action approach and habit theory. Solid lines indicate pathways tested in the present study where 1 = valuation pathway, 2 = prioritization pathway, 3 = habituation pathway, 4 = translation pathway, and 5 = inhibition pathway. PBC = perceived behavioral control.

The present research aims to offer a mechanistic account of the relationship between self-control and health-related intentions and behavior using RAA and habit constructs. Fig. 1 depicts the five pathways from self-control to health behavior that are tested here. Below, we review relevant research with respect to each pathway.

### The Valuation Pathway

According to Berkman et al. [28], “[t]here is nothing unique about self-control. Instead, decisions that we label self-control are merely a fuzzy subset of all value-based decisions, which involve selecting a course of action among several alternatives.” Self-control, in this view, is “no more and no less than value-based decision-making” (p. 423). Although Berkman et al. [28] favor a more process-oriented and neurobiologically-inspired account of value-based decision-making, the RAA captures many of the considerations specified in their account. Other research is also consistent with the idea that self-control influences how people appraise behaviors. Converse et al. [15] observed that trait self-control predicted the quality of participants’ motivation to act in four studies, and Hagger et al. [11] reported that self-control predicted attitudes, norms, and perceived behavioral control from the theory of planned behavior (TPB; [19]) in 6 datasets involving 10 health behaviors. TPB variables mediated the relationship between self-control and intentions, and intentions mediated the self-control—behavior relation. Thus, there is initial support for the valuation pathway from self-control to health behaviors.

### The Prioritization Pathway

Whereas the valuation route suggests that high self-control leads to stronger beliefs about the enjoyment or instrumental benefits of health behaviors, increased perceptions of normative support, and greater perceived ease of acting healthy, the prioritization pathway concerns how self-control influences the weight attached to these different considerations during intention formation. The valuation pathway is a mediation model (self-control → cognitions → intentions → behavior) whereas the prioritization pathway is a moderation model involving the hypothesis that higher self-control leads to improved prediction of intention by cognitive attitudes, perceived behavioral control, and injunctive norm and weaker relationships between intentions and affective attitudes, descriptive norms, and habits.

Self-control is associated with prioritizing long-term over short-term goals (e.g., [29–31]) and with appraising behaviors in a more detached, abstract, and “cool” manner [2, 32–34]. As cognitive attitudes generally reflect beliefs about the instrumental or long-term consequences of behavioral performance whereas affective attitudes index more immediate and visceral outcomes (e.g., [21]), higher self-control should lead to stronger associations between cognitive attitudes and intentions and weaker associations between affective attitudes and intentions. For the same reason, habits should have less influence on the process of intention formation when self-control is high. Self-control should also relate to improved prediction of intentions by perceived behavioral control. Giving appropriate weight to the feasibility of acting is adaptive for action control [35, 36], and there is evidence that self-control is associated with heightened sensitivity to the controllability of behaviors [37]. Finally, self-control is associated with the relative influence of injunctive vs. descriptive norms. Jacobson et al. [38] observed that an ego-depletion manipulation that lowered self-control led to a greater impact of descriptive norms but reduced the impact of injunctive norms (see also, [39, 40]). Jacobson et al. [38] proposed that self-control is needed to *resist* descriptive norms but it is needed to *comply* with injunctive norms. In sum, the prioritization pathway predicts that as self-control increases, cognitive attitudes, perceived behavioral control, and injunctive norms hold greater sway over intentions whereas affective attitudes, habits, and descriptive norms become less influential.

### The Habituation Pathway

Like the valuation pathway, the habituation pathway involves a mediation model but habits form the mediator here rather than attitudes, norms, or perceived behavioral control. The idea is that people with high self-control do not have to solely rely on effortful control of impulses to promote behavior change; they have also developed habits that can efficiently and effectively facilitate behavioral goals. Adriaanse et al. [13] offered the first test of the habituation pathway and found that habits indeed mediated the relationship between self-control and unhealthy snack consumption. Subsequent research by Galla and Duckworth [9] found that habits mediated the self-control-behavior relation for healthy eating, exercise, and sleep and, in a longitudinal study, habits explained the association between self-control and reaching meditation practice goals. Galla and Duckworth [9] suggested that high self-control enables people to organize situations and remove obstacles and thereby generating stable circumstances and behavioral repetition that promote habit formation.

### The Translation Pathway

The translation pathway concerns the hypothesis that self-control moderates the relationship between intentions and behavior such that higher self-control leads to improved translation of intentions into action. At least two mechanisms could underlie this pathway. First, self-control could be associated with more stable intentions, and research indicates that intention stability moderates intention-behavior consistency (e.g., [41, 42]). Second, self-control is associated with the propensity to form implementation intentions [43] which are an established moderator of intention-behavior relations [44]. Empirical support for the translation pathway came from Hagger et al. [11] who observed that intentions to restrict one’s diet were more strongly related to subsequent behavior when self-control was high as compared to low. The evidence was equivocal, however, as moderation of the intention-behavior relation was not observed for several behaviors and was even reversed for some behaviors (e.g., higher self-control was associated with weaker intention-behavior consistency for binge drinking). Further tests of the translation pathway thus are warranted.

### The Inhibition Pathway

The final, inhibition pathway involves the traditional conceptualization of self-control. As Fujita ([30], p. 355) pointed out, most scholars “explicitly or implicitly define self-control as the effortful control of impulses.” Moreover, the standard instrument used to index self-control, the Brief Self-Control Scale [3], includes several items that clearly index inhibition (e.g., “I am good at resisting temptation,” “I refuse things that are bad for me, even if they are fun,” “I have a hard time breaking bad habits”). In the present research, two variables qualify as possible impulsive factors, namely, affective attitudes and habits. That is, health behavior performance could be compromised by unwanted habits or affective influences. Thus, the hypothesis tested here is that self-control will moderate affective attitude-behavior and habit-behavior relations such that affective and habitual influence is weaker at higher levels of self-control.

### The Present Study

The foregoing discussion indicates that self-control could influence health behaviors via five routes: the valuation pathway (i.e., cognitions mediate the self-control-intention relation, and intention mediates the self-control-behavior relation); the prioritization pathway (i.e., self-control moderates the relationship between intentions and cognitive attitude, affective attitude, descriptive norm, injunctive norm, perceived behavioral control, and habit); the habituation pathway (i.e., habit mediates the association between self-control and behavior); the translation pathway (i.e., self-control moderates intention-behavior consistency); and the inhibition pathway (self-control moderates the relationship between behavior and affective attitudes and habits). So far as we can ascertain only one previous paper examined the valuation and translation pathways [11], two papers examined the habituation pathway [9, 13], and no field tests have been undertaken on the prioritization and inhibition pathways. Importantly, the present research offered the first *simultaneous* test of the five pathways. To offer a comprehensive test of these pathways, we examined eight different health behaviors among a large sample over a 3-month period and used multilevel modeling to test associations across behaviors. We examined both approach behaviors (i.e., health-protective actions such as physical activity and a low-fat diet) and avoidance behaviors (i.e., health-risk behaviors such as avoiding excess alcohol consumption and continuous sitting) and tested whether approach vs. avoidance behavior moderated each of the five pathways [1, 16].

## Method

### Respondents and Procedure

The research protocol was approved by the University of Leeds IRB. Participants were recruited via Prolific (https://www.prolific.co/) and rewarded £7.80 (~ $10) for completing questionnaires on two occasions about eight health behaviors (eating at least five portions of fruit and vegetables per day; undertaking recommended levels of physical activity each week; flossing teeth twice per day; eating a low-fat diet each day; avoiding eating unhealthy snack each day; avoiding drinking more than the recommended daily limits of alcohol; avoiding continuous sitting for more than 30 min at a time; avoiding eating more than two portions of red meat per week).

A total of 775 respondents completed the baseline questionnaire; 285 identified as male, 486 as female, 2 as non-binary and 2 preferred not to answer. In relation to ethnicity 664 identified as Caucasian, 14 as African, 19 as Latino/Hispanic, 35 as Asian, and 24 as another (unspecified) race. Mean age was 31.9 years (SD = 11.3) and mean socioeconomic status (SES) was 5.68 (SD = 11.3; assessed on a 1–10 ladder to represent standing in society with those at the top being people with the best off—those who have the most money, most education, and the best jobs). Three months later, 633 participants completed the second questionnaire and could be matched to baseline. This final sample size of 633 comfortably exceeds the minimum sample of 50 identified in modeling for multilevel modeling [45]. Data collection took place from September to December 2017.

### Measures

The wording of items followed recommendations for each construct [46]. We report the measures in relation to one example behavior (i.e., physical activity) below. In the baseline questionnaire, participants rated each of the eight behaviors on a measure of *affective attitude* (two items; e.g., “Taking the recommended levels of physical activity each week over the next three months would be: not enjoyable—enjoyable; unpleasant – pleasant”; median *r* = .84; scored 1–5 and averaged), *cognitive attitude* (two items; e.g., “Taking the recommended levels of physical activity each week over the next three months would be: worthwhile–pointless; unimportant–important”; median *r* = .73; scored 1–5 and averaged), *injunctive norms* (one item; e.g., ‘Most people important to me think that: I should–I should not: take the recommended levels of physical activity each week over the next three months, strongly disagree–strongly agree’; scored 1–5), *descriptive norms* (one item; e.g., “I think that most people who are important to me will take the recommended levels of physical activity each week over the next three months, strongly disagree–strongly agree”; scored 1–5), *perceived behavioral control* (two items; e.g., “If it were entirely up to me, I am confident that I could take the recommended levels of physical activity each week over the next three months, strongly disagree – strongly agree”; “How much control do you believe you have over taking the recommended levels of physical activity each week over the next three months, no control–complete control”; median *r* = .45; scored 1–5 and averaged), *behavioral intentions* (two items; e.g., “I am likely to take the recommended levels of physical activity each week over the next three months, strongly disagree – strongly agree”; “I intend to take the recommended levels of physical activity each week over the next three months, strongly disagree–strongly agree”; median *r* = .83; scored 1–5 and averaged) and *habit* (1 frequency of performance item [“I take the recommended levels of physical activity each week, never–always”], and 1 stability of context item [“Is taking the recommended levels of physical activity each week something that you would do at the same times and in the same places each time? definitely no–definitely yes”]). Both items were scored 1–5; habit was the multiplicative combination of the frequency and stability of context items [26, 47];). The habit measure was highly correlated with the frequency measure of past behavior (*r* = .842, *p* < .001). Participants also completed the 13-item Brief Self-Control Scale [3] (alpha = .85) (e.g., “I am good at resisting temptation, not at all–very much”; scored 1–5 and averaged, with higher scores indicating greater self-control). The Brief Self-Control Scale has high internal (*alpha* = .83–.85) and test–retest (*r* = .87 over a three week period) reliability [3].

At the three-month follow-up, the *behavior* was measured using three items per behavior that were standardized and averaged (e.g., “Over the past three months, how many weeks did you take the recommended levels of physical activity?, ____ weeks”, scored 0–12; “How frequently did you take the recommended levels of physical activity each week over the last three months? never–always”, scored 1–5; “Over the last three months, I took the recommended levels of physical activity each week, strongly disagree – strongly agree”, scored 1-–5; median alpha = .96). Only items relevant to the current research are reported here (the full questionnaire can be obtained from the first author).

### Analysis Plan

Data were analyzed in SPSS (version 20, SPSS Inc.) and HLM (version 7, SSI) using multi-level modeling. Participants who had missing data for the self-control measure or at least one variable missing for each behavior were excluded. Our data was organized into a two-level structure with affective attitude, cognitive attitude, injunctive norms, descriptive norms, perceived behavioral control, habit, behavioral intentions, and behavior at level 1 and self-control at level 2. For analyses involving behavioral intentions as the outcome, a total of 6,152 person-behavior data points spread across 775 individuals were used in the analysis. When behavior was the outcome, 4,513 person-behavior data points from 633 individuals were used in the analyses. Multi-level modeling allowed us to control for the fact that level 1 variables were assessed in relation to multiple behaviors within individuals. Consistent with our predictions, we assessed general patterns for variables across behaviors rather than focusing on individual health behaviors.

To test the *valuation* route, we first examined the correlations between RAA variables and self-control. Mediation was formally tested using the MLMED macro in SPSS developed by Rockwood at https://njrockwood.com/mlmed which allows for testing multi-level mediation effects. We tested whether affective attitude, cognitive attitude, injunctive norms, descriptive norms, and perceived behavioral control mediated the effects of self-control on behavioral intentions. The habit was also included in this mediation analysis to test the *habituation* pathway. We report the estimated direct and mediated effects and 95% confidence intervals.

We used Hierarchical Linear Modeling using HLM7 [48] to test the *prioritization* route. The behavioral intention was regressed on the level 1 variables (affective attitude, cognitive attitude, injunctive norms, descriptive norms, perceived behavioral control, and habit), the level 2 variable (self-control), and the cross-level interaction between the two. For each model, we report the deviance statistic. For each predictor, we report unstandardized coefficients and standard errors, standardized coefficients, and significance (all based on the population-average model with robust standard errors). When a cross-level interaction was significant, we explored simple slopes using Preacher’s software (http://www.quantpsy.org/interact/hlm2.htm; Model 3 for cross-level interactions). For each significant cross-level interaction, we also tested for differences between approach versus avoidance behaviors by testing the significance of the cross-level interaction after controlling for the approach versus avoidance behavior variable.

A further set of tests explored whether behavioral intentions, perceived behavioral control, habit, affective attitude, cognitive attitude, injunctive norms, and descriptive norms mediated the effects of self-control on behavior. Based on previous research showing intention, perceived behavioral control, and habit to be key direct predictors of behavior we examined these three variables as mediators in an initial model (Model 1) and then tested the addition of affective attitude, cognitive attitude, injunctive norms, and descriptive norms as additional mediators of the effects of self-control on behavior in a second model (Model 2). These mediation effects were assessed by examining simple correlations and the MLMED macro in SPSS.

To test the *translation* and *inhibition* routes, we used Hierarchical Linear Modeling in HLM7. In model 1, behavior was regressed on the level 1 variables (intentions, perceived behavioral control, habit), the level 2 variable (self-control), and the cross-level interactions between the two. In model 2, the behavior was regressed on the level 1 variables (intentions, perceived behavioral control, habit, affective attitude, cognitive attitude, injunctive norms, descriptive norms), the level 2 variable (self-control), and the cross-level interactions between the two. When interactions were significant, we examined simple slopes using the Preacher macro to interpret the moderation effects. For each significant cross-level interaction, we also tested for differences between approach vs. risk health behaviors by testing the significance of the cross-level interaction of predictor × approach versus avoid × self-control after controlling for approach versus avoidance behavior variable. To confirm the robustness of our findings, we reran the MLM analyses controlling for past behavior (instead of habit) and demographic variables that were significantly correlated with our key dependent variables (intentions and behavior). As habit and past behavior were correlated at *r* = .842, *p* < .001, multicollinearity precluded the entry of both these variables in the MLM analyses.

## Results

### Tests for Valuation Pathway

Table 1 shows the intercorrelations among self-control, habits, and RAA variables and also the mean and standard deviation for each variable. Self-control was positively correlated with behavioral intentions and each of the RAA predictors of intentions, except injunctive norms; RAA variables were also significantly correlated with intentions. These findings are consistent with the *valuation* pathway wherein self-control leads to setting more healthful goal intentions via affective attitudes, cognitive attitudes, descriptive norms, and perceived behavioral control. Multi-level mediation analysis tested the extent to which affective attitude, cognitive attitude, injunctive norms, descriptive norms, perceived behavioral control, and habit mediated the effects of self-control on intentions. As the MLMED macro program only permits simultaneous consideration of three mediators, we first entered each mediator independently and then considered the three strongest mediators together. Consideration of each mediator individually indicated mediation of the relationship between self-control and intentions by affective attitude (*B* = 0.222, SE = 0.024, *z* = 9.154, *p* <.001, 95%CI 0.176 to 0.271), cognitive attitude (*B* = 0.068, SE = 0.018, *z* = 3.764, *p* < .001, 95%CI 0.0341 to 0.1044), descriptive norms (*B* = 0.092, SE = 0.017, *z* = 5.383, *p* < .001, 95%CI 0.059 to 0.127), perceived behavioral control (*B* = 0.172, SE = 0.021, *z* = 8.352, *p* <.001, 95%CI 0.133 to 0.214) and habit (*B* = 0.223, SE = 0.024, *z* = 9.304, *p* <.001, 95%CI 0.177 to 0.270) but not injunctive norms (*B* = 0.017, SE = 0.013, *z* = 1.300, *p* = .194, 95%CI −0.009 to 0.043). When the three strongest mediators were considered simultaneously, there were parallel mediation effects for affective attitude (*B* = 0.128, SE = 0.016, *z* = 7.984, *p* < .001, 95%CI 0.098 to 0.162), habit (*B* = 0.128, SE = 0.016, *z* = 8.020, *p* < .001, 95%CI 0.099 to 0.161), and perceived behavioral control (*B* = 0.063, SE = 0.011, *z* = 5.609, *p* < .001, 95%CI 0.042 to 0.086); the direct effect of self-control on intentions remained significant (*B* = 0.048, SE = 0.025, *t* = 1.979, *p* = .048, 95%CI 0.0004 to 0.097). These findings are consistent with the *valuation* pathway.

**Table 1.** Descriptives and Intercorrelations for Reasoned Action Approach Variables, Habit, Behavioral Intentions, Behavior, and Self-Control

	AA	CA	IN	DN	PBC	H	BI	B	SC
Affective Attitude (AA)	–	0.477***	0.296***	0.376***	0.441***	0.496***	0.579***	0.392***	0.175***
Cognitive Attitude (CA)		–	0.512***	0.290***	0.300***	0.376***	0.502***	0.269***	0.072***
Injunctive norms (IN)			–	0.381***	0.159***	0.248***	0.319***	0.160***	0.020
Descriptive norms (DN)				–	0.312***	0.398***	0.447***	0.267***	0.114***
Perceived Behavioral Control (PBC)					–	0.540***	0.609***	0.403***	0.165***
Habit (H)						–	0.667***	0.473***	0.200***
Behavioral Intentions (BI)							–	0.539***	0.184***
Behavior (B)								–	0.238***
Self-Control (SC)									–
*Mean*	3.334	4.060	3.817	2.904	3.898	9.970	3.193	0.039	3.025
*SD*	1.248	1.138	1.094	1.288	0.947	7.233	1.404	0.907	0.726

*Note*. *** *p* < .001.

We then extended the test of the valuation route to behavior. All RAA predictors were significantly correlated with this outcome. Mediation tests for each individual predictor indicated that the self-control-behavior relation was mediated by intentions (*B* = 0.170, SE = 0.018, *z* = 9.494, *p* < .001, 95%CI 0.136 to 0.206), perceived behavioral control (*B* = 0.092, SE = 0.013, *z* = 7.025, *p* < .001, 95%CI 0.068 to 0.119), affective attitude (*B* = 0.120, SE = 0.015, *z* = 8.016, *p* < .001, 95%CI 0.092 to 0.151), cognitive attitude (*B* = 0.030, SE = 0.009, *z* = 3.473, *p* < .001, 95%CI 0.015 to 0.049), and descriptive norms (*B* = 0.040, SE = 0.009, *z* = 4.382, *p* < .001, 95%CI 0.023 to 0.059) but not injunctive norms (*B* = 0.0063, SE = 0.005, *z* = 1.245, *p* = .213, 95%CI −0.001 to 0.017). These findings offer clear support for the *valuation* pathway. The self-control-intention relation was mediated by RAA variables; intentions, and perceived behavioral control—the key determinants of behavior according to the RAA—both mediated the self-control behavior relation.

### Tests for Habituation Pathway

Consistent with the *habituation* pathway, self-control was correlated with both habit and behavior, and the habit was correlated with behavior. In a formal test, habit mediated the relationship between self-control and behavior (*B* = 0.126, SE = 0.016, *z* = 8.123, *p* < .001, 95%CI 0.097 to 0.159). To double check this conclusion, we undertook a simultaneous test of intentions, perceived behavioral control, and habits as these variables showed the strongest mediation of the self-control-behavior relation. MLMED analyses indicated that intentions, perceived behavioral control, and habits simultaneously mediated the association between self-control and behavior (*B* = 0.127, 0.030, and 0.037, SE = 0.017, 0.010, and 0.011, *z* = 7.691, 2.955, and 3.440, 95%CI 0.096, 0.162; 0.011, 0.051; and 0.016, 0.059, respectively, all *p*s < .001). Thus, findings supported both the valuation and habituation pathways.

### Tests for Prioritization Pathway

To test the *prioritization* pathway, we regressed intentions on RAA variables, habits, and self-control, and the cross-level interactions between self-control and each of these predictors (see Table 2). Findings showed significant positive interactions between self-control and both cognitive attitudes and perceived behavioral control and significant negative interactions between self-control and both injunctive norms and habits. Interactions between self-control and both affective attitudes and descriptive norms were not significant.

**Table 2.** Hierarchical Multi-Level Regression of Behavioral Intentions on Reasoned Action Approach Variables, Habit, Self-Control and Interactions (*N* of participants = 775; *N* of observations = 6,152)

Model	Predictors	*B*	SE	Beta
1.	Intercept (γ_00_)	3.192	.025	
	Affective Attitude (γ_10_)	0.159	.014	.137***
	Cognitive Attitude (γ_20_)	0.224	.016	.174***
	Injunctive norms (γ_30_)	0.047	.014	.037***
	Descriptive norms (γ_40_)	0.125	.013	.113***
	Perceived Behavioral Control (γ_50_)	0.422	.017	.305***
	Habit (γ_60_)	0.068	.003	.347***
	Self-Control (γ_01_)	0.366	.036	.186***
	Cross-level interactions with Self-Control			
	Affective attitude (γ_11_)	-0.024	.020	−.021
	Cognitive attitude (γ_21_)	0.053	.020	.041**
	Injunctive norms (γ_31_)	-0.039	.017	−.031*
	Descriptive norms (γ_41_)	-0.010	.016	−.009
	Perceived behavioral control (γ_51_)	0.068	.022	.049**
	Habit (γ_61_)	-0.010	.003	−.051**

*Note*. B = unstandardized coefficient; SE = standard error; Beta = standardized coefficient. Baseline intercept only model, deviance = 21751.3; model 1, deviance = 15938.2; *p < .05; ** *p* < .01; *** *p* < .001.

We decomposed the interactions via simple slope analyses at low (*M* – 1 SD) and high (*M* + 1 SD) levels of self-control. Findings showed that cognitive attitude better predicted intentions when self-control was high (*B* = 0.262, SE = 0.023, *p* < .001) as compared to low (0.185, SE = 0.021, *p* < .001; Supplemental Fig. S1). Similarly, perceived behavioral control was more strongly associated with intentions at high (*B* = 0.472, SE = 0.025, *p* < .001) vs. low levels of self-control (*B* = 0.373, SE = 0.022, *p* < .001; Supplemental Fig. S2). Conversely, injunctive norms and habits were weaker predictors of intention when self-control was high (*B* = 0.018 and 0.061, SE = 0.017 and 0.003, respectively, *p*s < .001) as compared to low (*B* = 0.075 and 0.076, SE = 0.020 and 0.004, respectively, *p*s < .001; Supplemental Figs. 3 and 4). These findings support the *prioritization* pathway. Higher self-control is associated with giving greater weight to cognitive attitudes and perceived behavioral control, and less weight to injunctive norms and habits during intention formation.

### Tests for Translation and Inhibition Pathways

The *translation* and *inhibition* pathways were tested via regressions of behavior on RAA variables, habits, and self-control, and the cross-level interactions between self-control and these predictors. Table 3 indicates that, as expected, self-control interacted with intention, habit, and affective attitudes. An unanticipated, negative interaction between self-control and the injunctive norm was also observed. Simple slopes analyses indicated that the predictive validity of intention increased as self-control moved from low (*B* = 0.132, SE = 0.021, *p* < .001) to high (*B* = 0.228, SE = 0.019, *p* < .001; Supplemental Fig. 5). Thus, the data supported the translation pathway. As self-control increased, intentions were more effectively translated into action.

**Table 3.** Hierarchical Multi-Level Regressions of Behavior on Reasoned Action Approach Variables, Habit, Self-Control and Interactions (*N* of participants = 633; *N* of observations = 4,513).

Model	Predictors	*B*	SE	Beta
1	Intercept (γ_00_)	−0.012	.020	
	Behavioral Intentions (γ_10_)	0.180	.014	.279***
	Perceived behavioral control (γ_20_)	0.071	.018	.079***
	Habit (γ_30_)	0.025	.002	.198***
	Self-control (γ_01_)	0.302	.027	.237***
	Cross-level interactions with self-control			
	Behavioral intentions (γ_11_)	0.066	.020	.102***
	Perceived behavioral control (γ_21_)	−0.039	.025	−.044
	Habit (γ_31_)	−0.007	.003	−.055*
2	Intercept (γ_00_)	−0.010	.020	
	Behavioral intentions (γ_10_)	0.177	.015	.274***
	Perceived behavioral control (γ_20_)	0.069	.018	.077***
	Habit (γ_30_)	0.023	.002	.182***
	Affective attitude (γ_40_)	0.037	.013	.033**
	Cognitive attitude (γ_50_)	−0.014	.016	−.012
	Injunctive norms (γ_60_)	−0.008	.014	−.007
	Descriptive norms (γ_70_)	−0.000	.015	−.007
	Self-control (γ_01_)	0.302	.027	.237***
	Cross-level interactions with self-control			
	Behavioral intentions (γ_11_)	0.074	.022	.115***
	Perceived behavioral control (γ_21_)	−0.025	.025	−.028
	Habit (γ_31_)	−0.007	.003	−.055*
	Affective attitude (γ_41_)	−0.037	.017	−.034*
	Cognitive attitude (γ_51_)	−0.019	.022	−.006
	Injunctive norms (γ_60_)	0.061	.019	.046**
	Descriptive norms (γ_70_)	−0.000	.015	−.046

*Note*. B = unstandardized coefficient; SE = standard error; Beta = standardized coefficient. Baseline Intercept only Model, Deviance = 11751.8; Model 1, Deviance = 10519.4; Model 2, Deviance = 10,443.5; + *p* < .10; *p < .05; ** *p* < .01; *** *p* < .001.

Decomposition of the interactions between self-control and affective attitude plus self-control and habit in predicting behavior revealed findings consistent with the inhibition pathway. Affective attitudes were not associated with behavior when self-control was high (*B* = 0.011, SE = 0.017, *p* = .546) but exhibited a significant association when self-control was low (*B* = 0.064, SE = 0.018, *p* < .001; Supplemental Fig. 6). Habits were less predictive of behavior when self-control was high (*B* = 0.019, SE = 0.003, *p* < .001) as compared to low (*B* = 0.030, SE = 0.004, *p* < .001; Supplemental Fig. 7). Although the self-control × injunctive norm interaction was significant, simple slopes analyses indicated that injunctive norms were not associated with behavior at either low (*B* = −0.0003, SE = 0.021, *p* = .989) or high levels of self-control (*B* = −0.028, SE = 0.024, *p* = .250; Supplemental Fig. 8).

Because four of the behaviors examined here involved approach responses (eating at least five portions of fruit and vegetables per day, undertaking recommended levels of physical activity each week, flossing teeth twice per day, eating a low-fat diet each day), and four involved avoidance responses (avoiding eating unhealthy snack each day, avoiding drinking more than the recommended daily limits of alcohol; avoiding continuous sitting for more than 30 min at a time, avoiding eating more than two portions of red meat per week), it was possible to compute an approach vs. avoidance behavior variable (approach = 1, avoidance = 0). We then tested whether approach vs. avoidance behavior influenced findings for five pathways tested here. There was little evidence that the type of behavior influenced the findings. For instance, approach vs. avoidance behavior did not moderate interactions between RAA variables, habits, and intentions (*p*s > .395) or interactions between RAA variables, habits, and behavior (*p*s > .567). Thus, the results supporting the valuation, prioritization, habituation, translation, and inhibition pathways do not appear to be qualified by whether approach vs. avoidance responses are at issue. Findings also remained the same when past behavior (instead of habit) and the two demographic variables that were correlated with intentions and behavior (ethnicity and socioeconomic status) were controlled.

## Discussion

Although self-control is reliably associated with health behaviors, relatively little research has been specifically concerned with understanding how or why self-control is related to behaviors. We integrated trait, reasoned action, and habit approaches to specifying five potential pathways from self-control to health behavior change, termed the *valuation, prioritization, habituation, translation,* and *inhibition* routes, and tested the pathways using longitudinal data over 3 months from more than 660 participants. The findings offer a rich picture of how self-control promotes health behaviors, one that extends far beyond that the traditional view of self-control as the mere effortful inhibition of impulses (e.g., [30],). First, consistent with Berkman et al.’s [28] analysis of “self-control as a value-based choice,” findings indicated that high self-control was associated with stronger beliefs in the utility of health behaviors (cognitive attitudes), greater expectations that health behaviors would bring enjoyment and pleasure (affective attitudes), increased perceptions of normative support for health actions (descriptive norms), and an enhanced sense of control over health behavior performance (perceived behavioral control). These RAA variables, in turn, predicted stronger behavioral intentions, and intentions predicted behavior. Formal mediation analyses confirmed that RAA variables mediated the self-control-intention relation and that intention and perceived behavioral control mediated the self-control-behavior relation. Thus, a key reason why self-control promotes health behaviors is that increased self-control leads people to see health actions as both desirable and feasible which, in turn, means that people set goals to undertake health-protective actions and to avoid health-risk behaviors and enact those goals.

Second, the present findings supported the hypothesis that self-control promotes habit formation which leads to higher rates of attainment of health goals [9, 13]. In a formal test, habits mediated the self-control to behavior relation. Adriaanse et al. [13] termed the habituation route “effortless self-control” as habits are automatic responses to situational cues and thus obviate the need for conscious, effortful guidance of behavior. The benefits of self-control for health behavior performance are thus at least partly a matter of “good” habits.

Third, self-control moderated intention-behavior consistency such that intentions were more effectively translated into action at higher levels of self-control. Importantly, these findings were obtained for eight health behaviors in relation to both approach and avoidance responses. Why did self-control benefit intention realization in the present study whereas Hagger et al. [11] observed significant self-control × intention interactions only for some behaviors in some samples? One explanation can be derived from Ajzen’s [49] analysis of the principle of compatibility. This principle holds that relations between broad personality traits (such as self-control) and subsequent action are most accurately assessed using aggregates of behavior rather than specific behaviors. Adopting a multilevel analysis of eight behaviors here accorded with this principle.

The present analyses of the valuation, habituation, and translation pathways offer strong tests that replicate and extend previous research on self-control and health behaviors. Importantly, however, we also obtained novel findings concerning the prioritization and inhibition pathways. Whereas the valuation route indicates that high self-control promotes the formation of healthful behavioral intentions, findings for the prioritization pathway show that self-control influences the basis of intention formation [50]. In particular, the intentions of people with high self-control are more strongly based on the instrumental consequences of health behaviors (cognitive attitudes) and the feasibility of acting (perceived behavioral control) than people with low self-control. Moreover, high self-control means that people are less reliant on habits and injunctive norms during intention formation. Thus, self-control not only influences how healthful are people’s intentions but also how people arrive at those healthful intentions.

These findings—that higher self-control means that behavioral decisions are more strongly rooted in concerns about utility and feasibility, and less embedded in prescriptions or habits—are consistent with evidence that self-control is associated with more abstract (vs. concrete) construal of action [30] and with heightened sensitivity to controllability considerations [37, 51]. However, the interactions between self-control and descriptive and injunctive norms observed here were not consistent with Jacobson et al.’s [38] proposal that self-control reduces the influence of descriptive norms but increases the influence of injunctive norms. In the present study, self-control did not moderate the relationship between descriptive norms and intentions, and the interaction between self-control and injunctive norm was negative, and not positive as Jacobson et al.’s [38] account predict. Two factors might explain these discrepancies. First, Jacobson et al [38]. manipulated rather than measured self-control using an ego-depletion task, and findings for state measures of self-control may be at odds with results obtained via trait measures [37]. Second, our finding showing that self-control leads to the weaker prediction of intentions by injunctive norms is actually consistent with Converse et al.’s [15] results indicating that self-control predicts autonomous reasons for acting. Acting on the basis of injunctive norms is the antithesis of autonomous motivation so injunctive norms should have less influence on goal setting at higher levels of self-control.

The present findings also offer some of the first evidence supporting the inhibition pathway in a field setting over an extended period. We observed that self-control moderated associations between behavior and both affective attitudes and habits such that anticipated affect and established routines had a weaker grip on subsequent behavior as self-control increased. Self-control thus enables people to overcome affective and habitual influences that could threaten health behavior performance. Two issues are notable here. First, it appears that self-control relates to affective attitudes and habits in two different ways. Self-control is not only associated with more positive affect toward health behaviors and stronger habits, but also moderates the influence of these variables. These findings would seem to speak to the idea that self-control promotes flexible action control—both promoting beliefs and routines that facilitate performance while simultaneously retaining the capacity to modify these predictors, if needed. Second, the present findings offer new insights into the strength of relations between behavior and affective attitudes and habits. Direct relations between affective attitudes and behavior, even controlling for intentions and perceived control, have been observed in several studies [52, 53]. We found that this direct relationship may depend upon the distribution of self-control scores in the sample, as affective attitudes did not predict behavior at high levels of self-control. The present demonstration that self-control moderates habit-behavior relations is also important as few studies have documented factors that diminish the predictive validity of habits [27].

The conceptual and empirical limitations of the present study should be acknowledged. At the conceptual level, we investigated five different pathways from self-control through RAA variables, and habits to health behaviors. Inevitably, participant burden, cost, and other considerations meant that we were unable to examine several additional variables that could contribute to these pathways. For instance, it would be valuable to integrate Converse et al.’s [15] analysis of the role of autonomous motivation in self-control effects with the 5-pathway model and test whether this variable contributes to the valuation pathway. Relatedly, it will be important for future research to discover whether self-control promotes intention realization by generating stable or well-formed intentions [27, 41, 42] or by facilitating the formation of implementation intentions [43]. It was also the case that only two impulsive factors, affective attitudes and habits, were examined here. Future studies should endeavor to include measures of automatic attitudes or approach bias and test whether self-control inhibits these implicit influences on health behaviors in field settings [54].

Although a longitudinal design permits strong inferences, the fact that we had only two timepoints, 3 months apart, meant that moment-by-moment changes in situational affordances and strategy use could not be captured. Ecological momentary assessment (EMA) methods (e.g., [55],) could offer a valuable complement to the design used here. EMA analyses could, for instance, test whether trait self-control is associated with ongoing deployment of situational strategies for self-control such as situation selection (e.g., avoiding temptations), reappraisal (e.g., reinterpreting tempting stimuli), or suppression (e.g., using willpower to overcome cravings) [29]. Relatedly, EMA could assess the extent to which social, economic, and physical circumstances (e.g., neighborhood walkability or safety, food “deserts”) facilitate or hinder health behavior performance. In addition, the use of only two timepoints meant that self-control and various proposed mediators were assessed at the same time point (T1). A three-timepoint design that assessed self-control, mediators and outcome (intention or behavior) at different time points would provide a stronger test of the five pathways tested here.

There are empirical limitations too. First, behavior was measured using self-reports. It was not feasible to obtain objective measures of the multiple behaviors examined here, and so replication of the present findings using non-reactive outcomes should be a priority for future research. Second, descriptive and injunctive norms were measured using single items and multi-item tests would be valuable to corroborate our findings. Third, we used single-item measures of one particular formulation (performance frequency × context stability) of habits. Future tests should also consider multi-item measures of habit and indices of perceived automaticity (see [56], for a review). A fourth limitation was that the study involved only eight behaviors and the follow-up period was only 3 months. Replicating the findings in the general population and clinical samples, and undertaking tests for other behaviors over longer periods would be desirable. A fifth limitation was that effect sizes on behavior could only be assessed through simple correlations given the limitations of multi-level modeling in this regard. The simple correlations reported in Table 1 indicate that while intentions had a large-sized effect on behavior (*r* > .5), while habit, affective attitudes and perceived behavioral control had medium-large sized effects (.3 < *r* < .5), and self-control and other variables had small-medium sized effects (.1 < *r* < .3).

Notwithstanding these limitations, the present research breaks new ground and has implications for future research. The analyses presented here constitute one of the first attempts to integrate trait, reasoned action, and habit approaches. Although the findings were largely consistent with Ajzen’s [49] proposal that personality traits exert their effects either by altering the value of RAA variables (i.e., the valuation pathway) or by altering the weight attached to RAA variables in predicting intentions and behavior (i.e., the prioritization and translation pathways), we also obtained support for additional pathways (i.e., the habituation and inhibition pathways). These findings have clinical and practical implications. The overall heritability of self-control is 60% [5] and accumulated research indicates that training is not effective at increasing self-control (e.g., [6, 57],). How then can health behavior change be promoted if self-control is low? Findings from the five pathways tested here help to answer this question. As self-control leads to weaker attitudes, norms, perceived behavioral control, and intentions, persuasive communication and other behavior change techniques could be used to change these cognitions (see [58, 59], for reviews). Relatedly, implementation intentions could be used to alter the weight attached to different considerations during intention formation [60], undermine the predictive validity of the effect and habits (e.g., [61, 62]), and promote the translation of intentions into action (e.g., [44]). Thus, the present integration of trait, reasoned action, and habit approaches indicate that low self-control is not an insurmountable barrier to behavior change. There are a variety of techniques that behavioral medicine researchers can use to ensure that low self-control does not undermine people’s efforts to engage in health behaviors.

## Supplementary Material

kaac053_suppl_Supplementary_Figures
