# Supplementary material for: How Does Self-Control Promote Health Behaviors? A Multi-Behavior Test of Five Potential Pathways
Source: Ann Behav Med. 2022 Oct 7;57(4):313–22. doi: 10.1093/abm/kaac053 (PMC10094954; doi:10.1093/abm/kaac053)
Supplement: kaac053_suppl_Supplementary_Figures [file kaac053_suppl_supplementary_figures.docx]

Supplemental Figure 1. Simple slopes for relationship between cognitive attitude and behavioral intention at low (M-1SD) and high (M+1SD) levels of self-control. Note that plotted values are between 1SD below (-1) and above (1) the predictor on the x-axis.

Supplemental Figure 2. Simple slopes for relationship between perceived behavioral control and behavioral intention at low (M-1SD) and high (M+1SD) levels of self-control. Note that plotted values are between 1SD below (-1) and above (1) the predictor on the x-axis.

Supplemental Figure 3. Simple slopes for relationship between injunctive norms and behavioral intention at low (M-1SD) and high (M+1SD) levels of self-control. Note that plotted values are between 1SD below (-1) and above (1) the predictor on the x-axis.

Supplemental Figure 4. Simple slopes for relationship between habit and behavioral intention at low (M-1SD) and high (M+1SD) levels of self-control. Note that plotted values are between 1SD below (-1) and above (1) the predictor on the x-axis.

Supplemental Figure 5. Simple slopes for relationship between behavioral intention and behavior at low (M-1SD) and high (M+1SD) levels of self-control. Note that plotted values are between 1SD below (-1) and above (1) the predictor on the x-axis.

Supplemental Figure 6. Simple slopes for relationship between affective attitude and behavior at low (M-1SD) and high (M+1SD) levels of self-control. Note that plotted values are between 1SD below (-1) and above (1) the predictor on the x-axis.

Supplemental Figure 7. Simple slopes for relationship between habit and behavior at low (M-1SD) and high (M+1SD) levels of self-control. Note that plotted values are between 1SD below (-1) and above (1) the predictor on the x-axis.

Supplemental Figure 8. Simple slopes for relationship between injunctive norms and behavior at low (M-1SD) and high (M+1SD) levels of self-control. Note that plotted values are between 1SD below (-1) and above (1) the predictor on the x-axis.
